# “Inhibitory effect of Brazilian red propolis on *Candida* biofilms developed on titanium surfaces”

**DOI:** 10.1186/s12906-020-02893-9

**Published:** 2020-04-03

**Authors:** Loyse Martorano-Fernandes, Yuri Wanderley Cavalcanti, Leopoldina de Fátima Dantas de Almeida

**Affiliations:** 1 Postgraduate Program in Dentistry, Federal University of Paraíba, Cidade Universitária, João Pessoa, Paraiba Brazil; 2 Department of Clinic and Social Dentistry, Federal University of Paraíba, Cidade Universitária, João Pessoa, Paraiba Brazil

**Keywords:** Biofilms, *Candida*, Peri-implantitis, Infection control

## Abstract

**Background:**

Peri-implant inflammation resulting from the presence of *Candida* biofilms may compromise the longevity of implant-supported dentures. This study evaluated the inhibitory effect of Brazilian red propolis on mono-species biofilms of *C. albicans* (ATCC 90028) and co-culture biofilms of *C. albicans* (ATCC 90028) and *C. glabrata* (ATCC 2001), developed on titanium surfaces.

**Methods:**

Titanium specimens were pre-conditioned with artificial saliva and submitted to biofilm formation (1 × 10^6^ CFU/mL). After 24 h (under microaerophilic conditions at 37 °C) biofilms were submitted to treatment for 10 min, according to the groups: sterile saline solution (growth control), 0.12% chlorhexidine and 3% red propolis extract. Treatments were performed every 24 h for 3 days and analyses were conducted 96 h after initial adhesion. After that, the metabolic activity (MTT assay) (*n* = 12/group), cell viability (CFU counts) (*n* = 12/group) and surface roughness (optical profilometry) (*n* = 6/group) were evaluated. Data from viability and metabolic activity assays were evaluated by ANOVA and Tukey tests. Surface roughness analysis was determined by Kruskal Wallis e Mann Whitney tests.

**Results:**

Regarding the mono-species biofilm, the cell viability and the metabolic activity showed that both chlorhexidine and red propolis had inhibitory effects and reduced the metabolism of biofilms, differing statistically from the growth control (*p* < 0.05). With regards the co-culture biofilms, chlorhexidine had the highest inhibitory effect (*p* < 0.05). The metabolic activity was reduced by the exposure to chlorhexidine and to red propolis, different from the growth control group (*p* < 0.05). The surface roughness (Sa parameter) within the mono-species and the co-culture biofilms statistically differed among groups (p < 0.05).

**Conclusions:**

Brazilian red propolis demonstrated potential antifungal activity against *Candida* biofilms, suggesting it is a feasible alternative for the treatment of peri-implantitis.

## Background

Peri-implantitis is a biofilm-dependent disease, in which the presence of bacteria and fungi within peri-implant tissues results in an inflammatory response [1]. Deficient hygiene, alcohol and tobacco consumption, systemic conditions including diabetes and immunosuppression can modulate peri-implantitis [2]. Multispecies biofilms frequently involved with peri-implantitis include proteolytic bacteria, such as *Porphyromonas gingivalis* and *Prevotella intermedia* [3, 4], in association with saccharolytic bacteria (*Streptococcus mutans* and *Streptococcus sanguinis*) and fungal species (*Candida* sp) [2, 5, 6].

In this context, 30% of the microorganisms identified in peri-implant biofilm are fungi of the genus *Candida* [7]. The presence of those microorganisms, in association with deficient hygiene, can induce a harmful microenvironment, in which the multispecies communities can release toxins and by-products that makes treatment unsuccessful [8]. Therefore, some clinical procedures have been proposed, including abrasive therapies with air, water, sodium bicarbonate, citric acid, plastic curettes scraping, ultrasonic cleansing, among others [9, 10]. However, none of those methods have obtained satisfactory results [8].

The use of topical antimicrobial agents associated with standard cleansing methods has been suggested [11]. In fact, antimicrobial mouthwash solutions are the most widespread, because it is an easy and cheap method [11]. Chlorhexidine 0.12% is considered the chemical gold standard for the treatment of peri-implantitis [12]. Although its use is recommended, using the therapy for a prolonged period of time has adverse effects, such as desquamated lesions, teeth and mucosal staining, loss of taste and dry mouth [13]. Therefore, the search for agents that present antimicrobial potential; with fewer side effects has been observed. Bioactive molecules and natural products have been investigated with regards to their potential to interfere with the adhesion and proliferation of microorganisms [14, 15].

In this context, the Brazilian red propolis has demonstrated an inhibitory effect against mono-species biofilm of *C. albicans* in a similar manner to that of chlorhexidine [16–18]. Propolis is a natural resin produced by bees [19], with antitumor, anti-oxidative and antimicrobial activities [20]. Its effects are due to the presence of flavonoids within the extracts, such as quercetin, rutin and kaempferol [21]. Thereby, Brazilian red propolis is suggested for treatment of biofilm-dependent diseases, in both forms of mouthwash and denture cleanser.

However, the use of red propolis extract has not been evaluated under peri-implantitis-like microenvironment. Thus, the aim of this study was to evaluate the inhibitory effect of the Brazilian red propolis on mono-species biofilms of *C. albicans* (ATCC 90028) and co-culture biofilms of *C. albicans* (ATCC 90028) and *C. glabrata* (ATCC 2001), developed on titanium surfaces. Our findings suggest that extract may be used for peri-implantitis disease, but cytotoxic effects could be a limitation for clinical applications.

## Methods

### Microbial strains and growth conditions

Strains of *C. albicans* (ATCC 90028) and *C. glabrata* (ATCC 2001) were cultivated aerobically on Sabouraud Dextrose (Merck KGaA, Germany) agar at 37 °C. Cell suspensions were grown in RPMI 1640 (Inlab diagnóstica, Brazil) during 24 h at 37 °C. Before experiments, cells were centrifuged (5000 *g* for 5 min), washed twice with sterile saline, and suspended in RPMI 1640 medium. Suspensions were standardized at OD_600_ = 1.0 (1 × 10^6^ cells/mL) (LGL Scientific 0741/16, Brazil), based in experiments described previously with some adaptations [22].

### Specimens’ preparation

Commercially pure titanium discs (1.3 × 0.2 cm) were prepared and polished in a barrel with abrasive paste and ceramic particles for 8 h [23]. Subsequently, they were cleaned with 70% alcohol (v/v) and sterilized by autoclave at 121 °C for 15 min. Specimens were allocated into three different groups (*n* = 12/group). Hydroalcoholic extract of Brazilian red propolis at 3% concentration (v/v) was used as experimental group. Chlorhexidine at 0.12% (Colgate-Palmolive, São Paulo, Brazil) was used as positive control, whilst sterile saline solution was used as growth control.

### Salivary pellicle formation and biofilm development

Initially, salivary pellicle was induced by immersing specimens in artificial saliva composed of 1% carboxymethyl (w/v); 0.0084% sodium chloride (w/v); 0.12% potassium chloride (w/v); 0.0342% potassium phosphate (w/v); 0.0146% calcium chloride (w/v), and 0.052% magnesium chloride (w/v) [24] and incubated at 37 °C for 60 min. Afterwards, the specimens with salivary pellicle were transferred to 24-well plates.

Microbial cell suspensions in RPMI 1640 medium (1 × 10^6^ cells/mL) were added together with RPMI 1640 medium (10× dilution) to generate mono-species biofilm of *C.albicans* and co-culture biofilm of *C.albicans* and *C.glabrata* on the surface of titanium discs. Plates were then was incubated for 24 h at 37 °C, under micro-aerobic conditions. The micro-aerobic atmosphere was generated by using an anaerobic jar with a candle, which reduced the presence of oxygen, similar to a peri-implant-like environment. After 24 h incubation, unattached cell suspension was aspirated. Specimens were then exposed to substances and washed twice with saline. Culture medium was renewed every 24 h, after each treatment.

### Expositions to tested substances

The hydroalcoholic extract of Brazilian red propolis (Laboratory Edimel, Paraíba, Brazil) was obtained at initial concentration of 30% (w/v) and diluted in sterile saline to yield concentration of 3% (w/v), as determined previously [16]. The sterile saline was used as growth control of biofilm. The specimens were exposed to the solutions at 24 h, 48 h and 72 h from the start of biofilm formation. In each exposition episode, specimens were immersed in tested substances and remained in contact with them for 10 min. After exposition, specimens were washed twice with sterile saline solution and the culture medium was renewed, followed by incubation under micro-aerobic conditions at 37 °C. Analyses were made at 96 h (4 days).

### Cell viability analysis

For cell viability analysis, the specimens were transferred to tubes containing 1.0 mL sterile saline solution, submitted to agitation in a vortex for 60 s, followed by serial dilution of the aliquots to determine the number of viable microorganisms (10^− 1^ up to 10^− 6^).

Aliquots of 10 μl of each serial dilution were seeded in triplicate on Sabouraud dextrose agar. The plates were then incubated at 37 °C for 48 h for later viable colony counts reading. The number of viable cells was counted and the values multiplied by the serial dilution. Data was expressed in colony forming unit per milliliter (CFU/mL).

### Cell metabolism assay

Cell metabolism was analyzed by means of MTT (methyl-tretrazolium salt) assay. For this, specimens were incubated with 600 μL of culture medium containing 10% of MTT (Sigma-Aldrich, St. Louis, MO, USA). The salt was oxidized by the SDH (succinate dehydrogenase) enzyme present in the respiratory chain of the fungi, and then used to determine the viable cell metabolism. Specimens were incubated at 37 °C for 3 h in the presence of MTT salt and the system was protected from the light. After this, the supernatant was removed and 600 μL isopropanol acid (6 N-HCl) (Sigma-Aldrich, St. Louis, MO, USA) was inserted, followed by sample homogenization. The supernatant was collected and its absorbance analyzed under spectrophotometer at 570 nm (LGL Scientific 0741/16, Brazil).

### Surface roughness

The surface roughness of the biofilms (*n* = 6/group) was determined by means of Profilometer analysis (CCI MP, Taylor Hobson, England). This analysis aimed to assess the presence and complexity of biofilms. More complex and thicker biofilms also present greater surface roughness. The biofilms were fixed in an aqueous solution of 2.5% glutaraldehyde (Sigma-Aldrich, St. Louis, MO, USA), at 4 °C for 24 h, and dehydrated at room temperature through increasing cycles of ethanol (50 to 100%). Surface roughness measurements (μm) were taken at two distinct points of the specimen, under 20× magnification, considering the measurement standards xy (1024 × 1024 pixels), xyz (512 × 512 pixels) and z (256 × 256 pixels). The speed of 3× was established, being the Surface Roughness Area (Sa) explored. Specimens without any biofilm (baseline) on the surface were also evaluated and used as reference for the analysis of developed biofilms.

### Data analysis

Data from viability, metabolic activity and surface roughness anslyses were evaluated with regards their normality and homocedasticity. Logarithmic transformation of viable cell counts was performed for statistical purposes. Statistical analysis was performed by means of one-way analysis of variance (ANOVA) and Tukey tests. All analyses were performed with 5% significance and power of 80%.

## Results

**Fig. 1:**
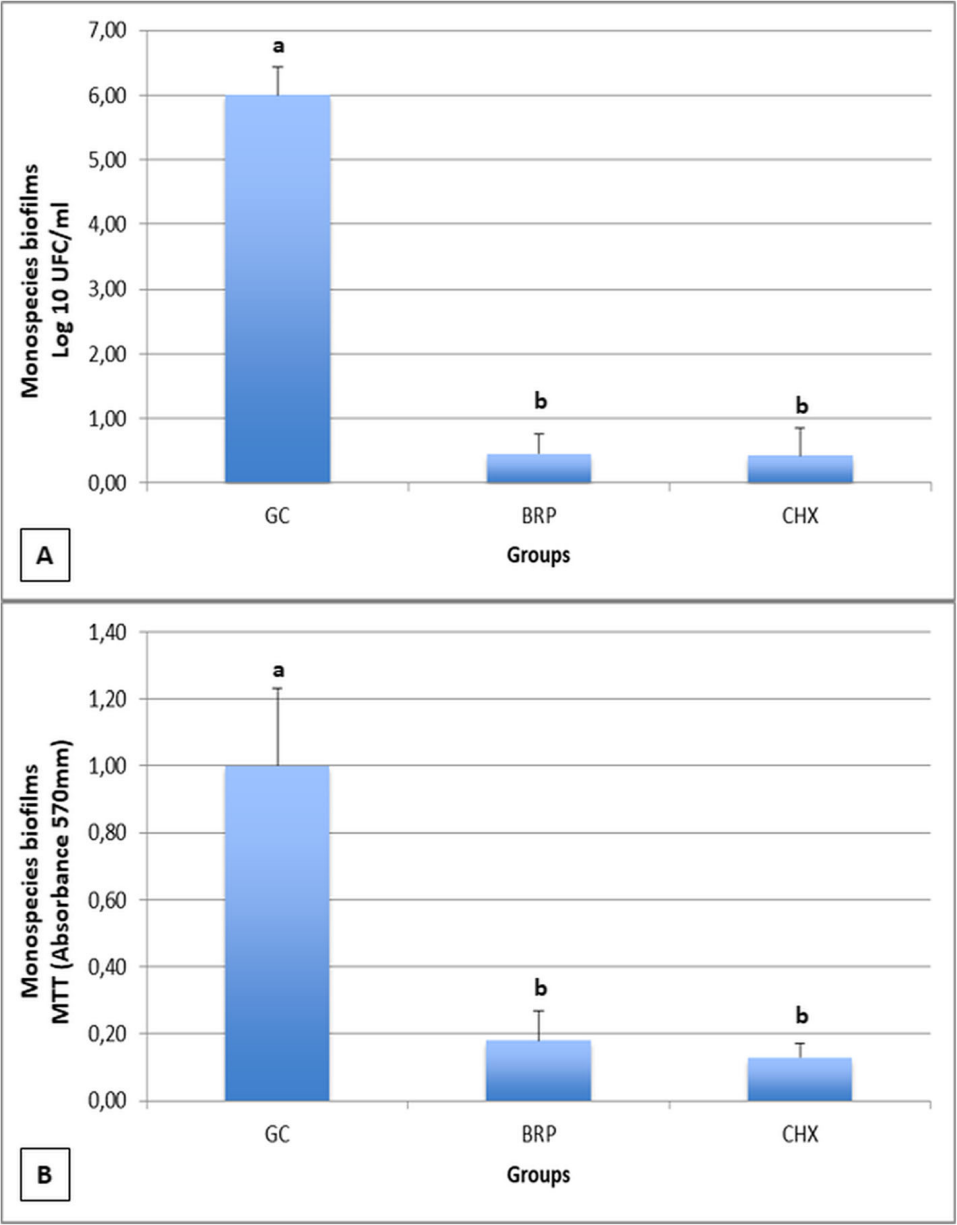
**a** Cell viability of biofilms (UFC/mL). Biofilms of *C. albicans* exposed to saline were considered as growth control. Columns represent averages and error bars represent standard deviations (*n* = 12). **b** Cell metabolism by the MTT assay. Biofilms of *C. albicans* exposed to saline were considered as growth control. Columns represent averages and error bars represent standard deviations (n = 12). Groups identified with the same letter do not differ statistically (Tukey, *p* > 0.05). GC: growth control; BRP: Brazilian red propolis; CHX: chlorhexidine

Cell viability determined that both chlorhexidine and red propolis extract had inhibitory effects on the proliferation of mono-species biofilms of *C. albicans*, differing statistically from the growth control (*p* < 0.05) (Fig.1a). With regards to the metabolic activity of biofilms, both chlorhexidine and red propolis reduced the metabolism of mono-species biofilms of *C. albicans*, differing statistically from the growth control (*p* < 0.05) (Fig.1b). The results of viability and metabolic activity in mono-species biofilms of *C. albicans* showed that Brazilian red propolis extract presented similar effect to that observed for chlorhexidine (*p* > 0.05).

**Fig. 2:**
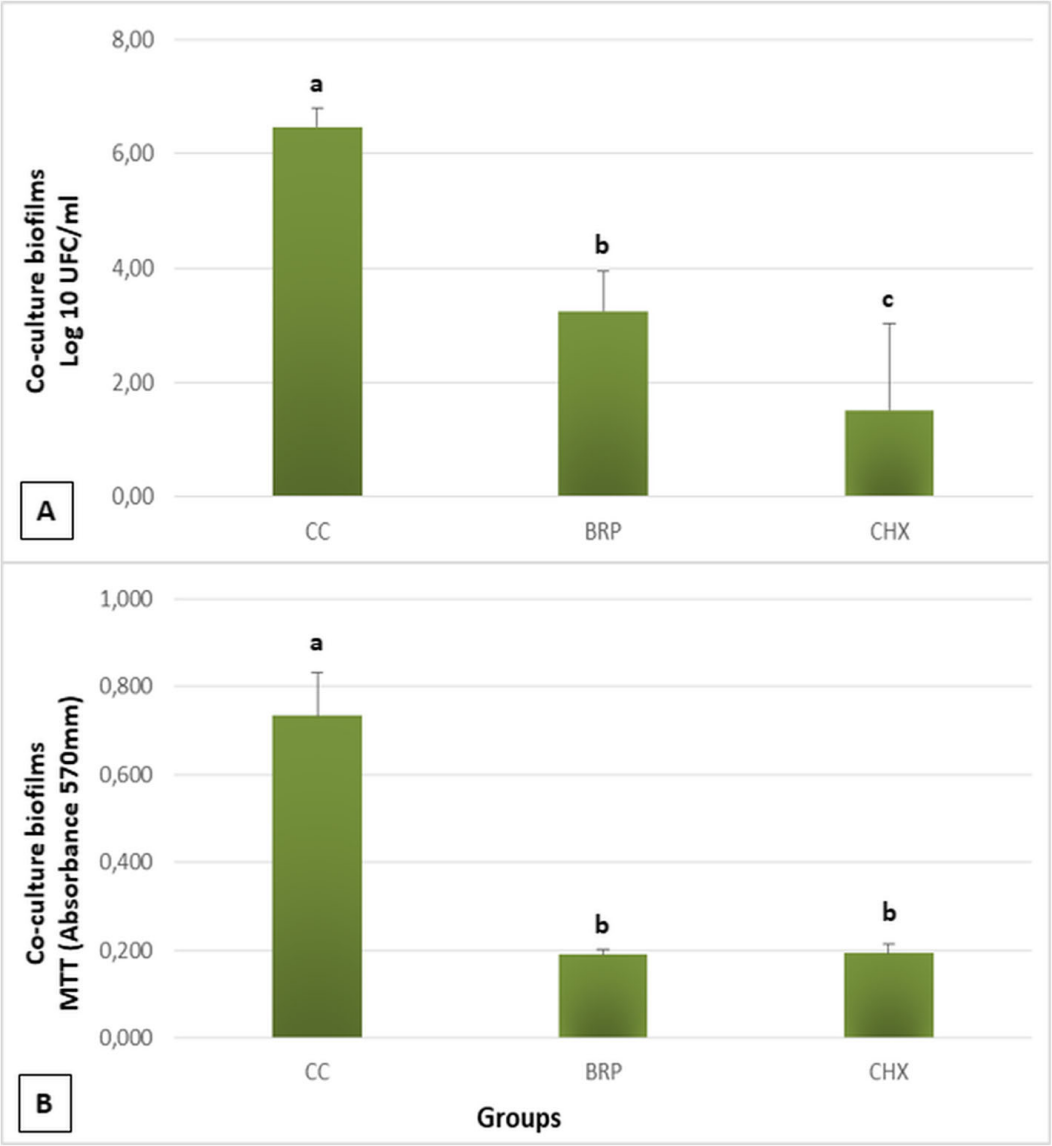
**a** Cell viability of biofilms (UFC/mL). Biofilms of *C. albicans* and *C. glabrata* exposed to saline were considered as growth control. Columns represent averages and error bars represent standard deviations (*n* = 12). **b** Cell metabolism by the MTT assay. Biofilms of *C. albicans* and *C. glabrata* exposed to saline were considered as growth control. Columns represent averages and error bars represent standard deviations (n = 12). Groups identified with the same letter do not differ statistically (Tukey, *p* > 0.05). GC: growth control; BRP: Brazilian red propolis; CHX: chlorhexidine

Within the co-culture biofilms, chlorhexidine had the highest inhibitory effect, differing from the other substances (*p* < 0.05) (Fig.2a). Although Brazilian red propolis extract did not inhibited co-cultures biofilm as chlorhexidine, there was a considerable inhibitory effect, statistically different from the growth control (*p* < 0.05). The exposure to chlorhexidine and to Brazilian red propolis extract reduced significantly the metabolism of co-cultured biofilms (Fig. 2b) (*p* < 0.05).

**Fig. 3:**
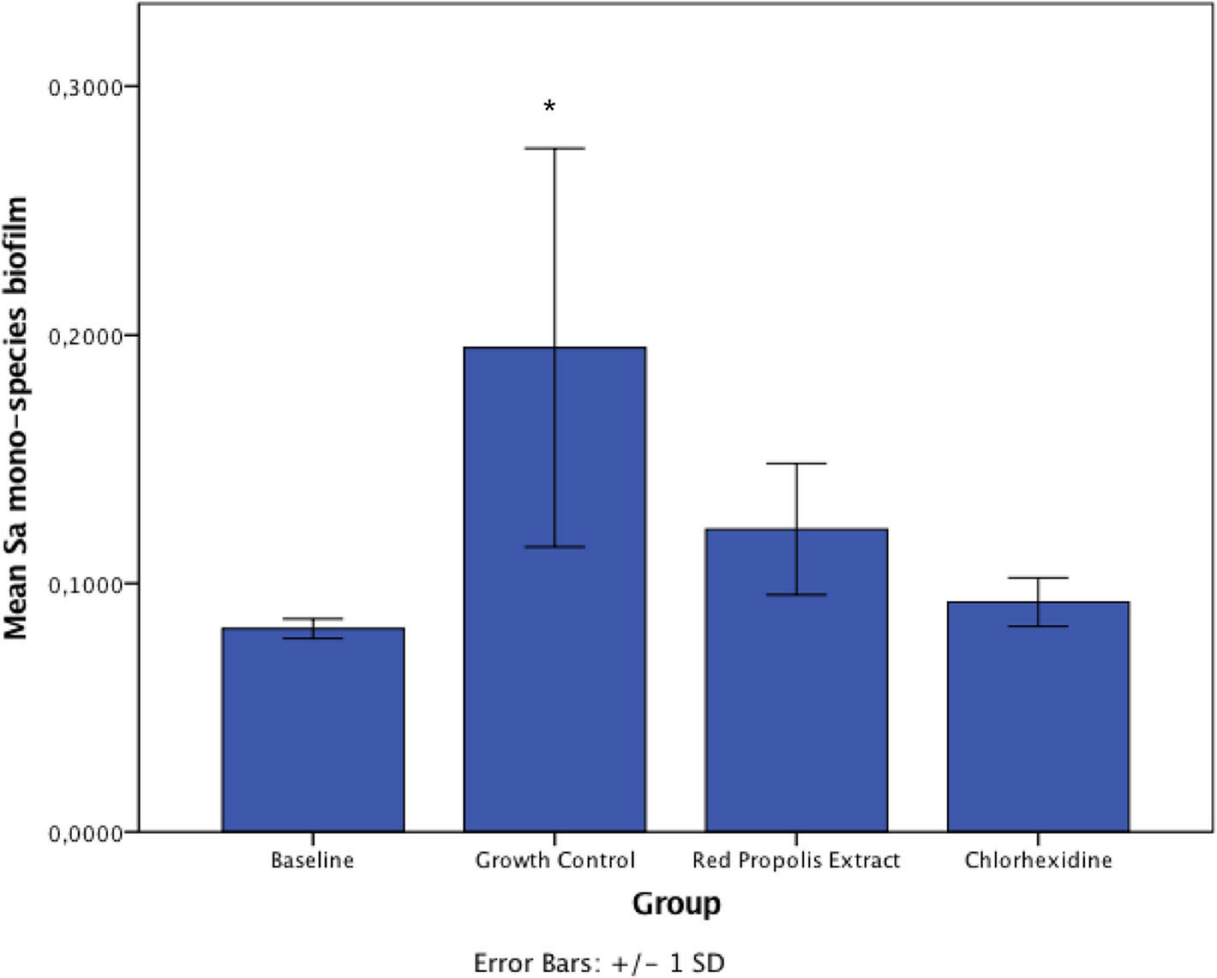
Comparison of Surface Roughness Area (Sa) among mono-species biofilms of *C. albicans* treated with the substances (*n* = 6/group). Asterisk shows that growth control presented significantly higher surface roughness compared to other groups (*p* < 0.05). This is suggestive that chlorhexidine and red propolis extract removed most of biofilm, similarly to clean (baseline) specimens

**Fig. 4:**
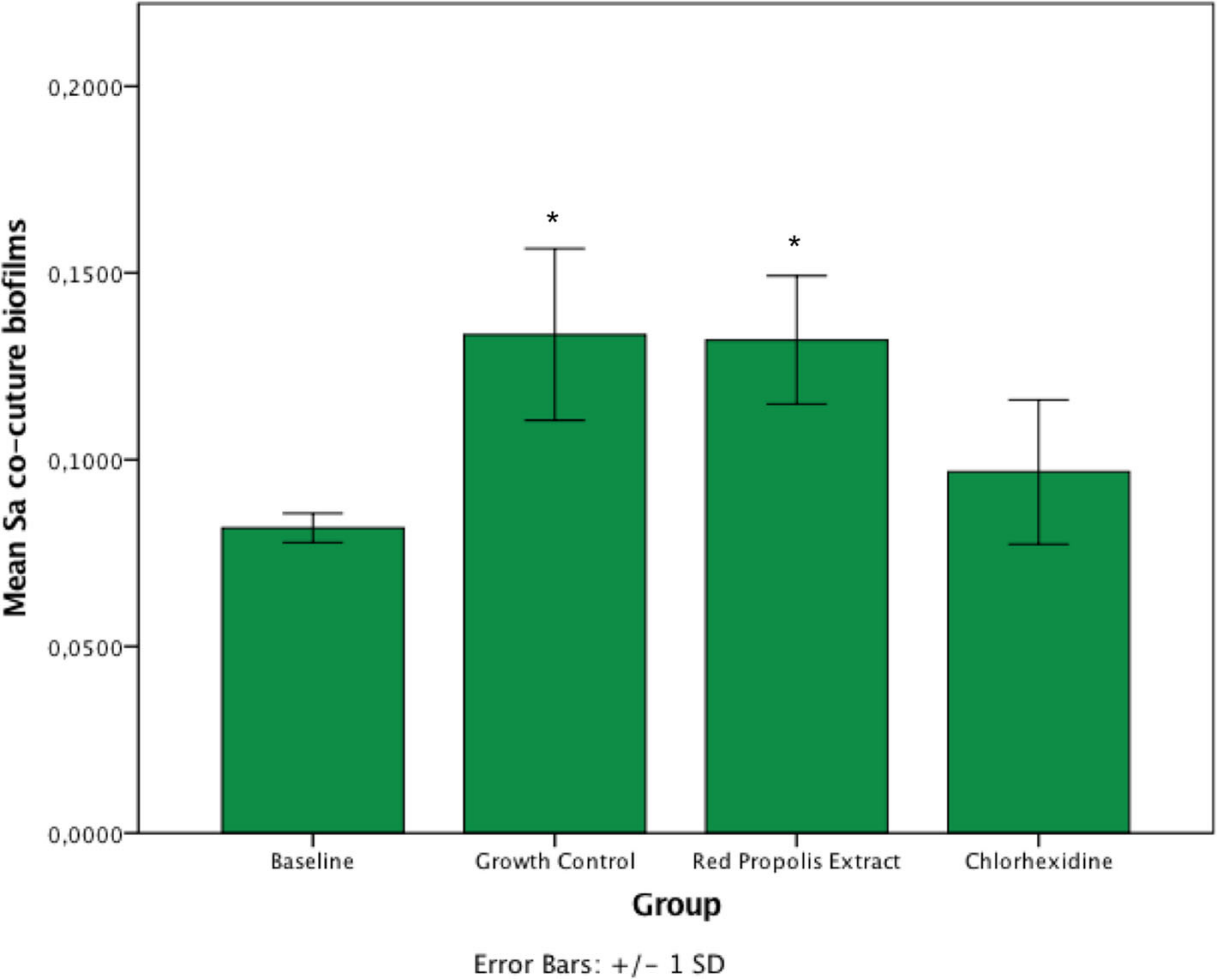
Comparison of Surface Roughness Area (Sa) among co-culture biofilms treated with the substances (*n* = 6/group). Asterisks show that growth control and red propolis extract presented significantly higher surface roughness compared to other groups (*p* < 0.05). This is suggestive that chlorhexidine removed most of biofilm, similarly to clean (baseline) specimens

The surface roughness is indicative of the presence of biofilm. In our study, the baseline (specimens without biofilm) was used for comparison with all treatment groups. Within mono-species biofilms of *C. albicans*, the surface roughness of specimens treated with chlorhexidine and red propolis extract did not differ from baseline (*p* > 0.05), but statistically differed from control (*p* < 0.05) (Fig. 3). This suggests treatments significantly reduced the presence of biofilm. With regards to co-culture biofilms, specimens treated with chlorhexidine did not differ from baseline (*p* > 0.05), but statistically differed from control and red propolis extract (*p* < 0.05) (Fig. 4). This suggests red propolis extract was less effective than chlorhexidine within co-culture biofilms.

## Discussion

Different treatment protocols for peri-implantitis have been studied, including chemical and mechanical intervention strategies [10, 25, 26]. Considering the increasing use of natural products, we have show that Brazilian red propolis hydroalcoholic extract have inhibitory effect on mono-species biofilms of *Candida albicans* and co-culture biofilms of *C. albicans* and *C. glabrata* developed on titanium surfaces. Results from this study show that Brazilian red propolis extract has similar antimicrobial activity of chlorhexidine-based mouthwash. Our findings may be useful for the biofilm control of implant-supported dentures.

The use of antimicrobial topical agents may be effective and well-disseminated method to prevent biofilm accumulation, especially for regions that are difficult to clean, such as peri-implant sites. Although chlorhexidine have been extensively used for biofilm control, its toxic potential to the mucosa contra-indicates its use for prolonged periods [13]. Brazilian red propolis hydroalcoholic extract at 3% has demonstrated a cytotoxic activity around 43% against fibroblasts monolayers (L-929) [16]. It is worth emphasizing that the implementation of an antimicrobial therapy must modulate the cell proliferation, so that when it debilitates the infection, there must not be great damage to the subjacent tissue. For this reason, Brazilian red propolis extract were selected according to the minimum inhibitory concentration (MIC) previously reported within the literature [16].

The 3% red propolis hydroalcoholic extract demonstrate an inhibitory effect on mono-species biofilm of *C. albicans* in a manner similar to that of chlorhexidine. Propolis is a natural resin produced by bees [19], and has a varied chemical composition depending on to geographic location, bee species and season of the year [27]. Diverse formulations of propolis have been evaluated with regards their antitumoral, anti-oxidative and antimicrobial activity [20], being these effects acknowledged to flavanoids present within the extracts [21]. Flavonoids also have anti-inflammatory activity, which is an appropriate characteristic for a mouthwash [28]. Further studies however are necessary to assess toxicity to cell lines and tissue culture, in order to give support to future clinical investigations [29].

The antimicrobial activity of Brazilian red propolis, in particular, has been determined against microorganisms such as *Staphylococcus spp*, *Actinomyces naeslundii* and gram-negative, such as *Pseudomonas aeruginosa* and *Salmonella typhimurium* [20, 30]. Moreover, their inhibitory activity has been reported against planktonic cultures of *Streptococcus mutans*, *Streptococcus sobrinus*, *Staphylococcus aureus*, *Actinomyces naeslundii* [17, 18]. We observed that the cell viability (CFU/mL) in mono-species biofilms of *C. albicans* was affected by exposure to the 3% (w/v) red propolis hydroalcoholic extract. However, the red propolis extract didn’t demonstrate the same efficacy against co-culture biofilms. Probably, the potential effect of the Brazilian red propolis extract would be superior in less complex biofilms with lower capacity for cell adhesion and proliferation, and therefore with greater transport of antimicrobial substances along the water channels and extracellular matrix.

Popular medicine has used propolis extract to treat throat infections and many commercial products are available nowadays varying their concentration from 10 to 30% (w/v). In the present study, we have used a concentration equivalent to 10× dilution of a 30% (w/v) commercial hydroalcoholic extract, which could be used to prepare a homemade mouthwash. Therefore, it should be considered that the effect evaluated in the present study consisted of a diluted extract available commercially, or a possible dilution in the mouth after swallowing (3× dilution of a 10% (w/v) product).

Our findings corroborate those of authors who have demonstrated the inhibitory effect of this substance against species of *Candida* in patients with periodontitis [31, 32], and using in a multispecies biofilm of *Staphylococcus aureus*, *Staphylococcus epidermides*, *Pseudomonas aeruginosa*, *Candida albicans*, *Candida tropicalis* and *Cryptococcus neoformans* [31]. Although a promising antimicrobial activity has been demonstrated for Brazilian red propolis extract, further studies are necessary for identifying which of the phytochemicals are involved with the antimicrobial activity.

The concentration of flavonoids such as quecertin, rutin and kaempferol can vary according to the period of collection of crude material. Based on that, variations on the antimicrobial activity of red propolis extract might be observed [19]. However, there are no findings that could confirm the same results against *Candida* species. Although clinical trials are necessary, this substance was shown to be an effective alternative for the treatment of *Candida* infections. In this study, the Brazilian red propolis hydroalcoholic extract at 3% concentration presented similar results to that observed for chlorhexidine, for both mono-species of *C. albicans* and co-culture biofilms of *C. albicans* and *C. glabrata*. Literature has reported that red propolis extract also has anti-oxidant effects, similarly to green propolis extracts, depending on concentration [33]. The bioactivity of Brazilian red propolis extract by using in vitro models of oral epithelium is strongly suggested.

The surface roughness observed for biofilms treated with red propolis hydroalcoholic and chlorhexidine suggests that these treatments had equivalent efficacy. Stronger evidence of similar efficacy of red propolis and chlorhexidine is due to the reduction of cell viability and metabolic activity of biofilms. The presence of remaining biofilms even after successive processes of cleaning with antimicrobial solutions is an aspect that has been observed in various studies [34–36]. Based on that, the literature has demonstrated that chemical cleansing solutions are not completely efficient in sterilizing surfaces; however, they contribute to reducing the microbial load. Under the conditions of the present study, the Brazilian red propolis extract reduced the *Candida* biofilm load and activity. This shows a positive aspect because there is lower risk of causing biofilm imbalance in vivo, with proliferation of opportunist species. Therefore, we suggest that the daily cleansing protocols evaluated in the present manuscript contribute to the oral hygiene of individuals with dental implants.

Future studies must consider an even more complex multispecies biofilm, with the involvement of other microbial species related to the etiology of peri-implantitis. However, to evaluate the effect of antimicrobial agents, the composition of multispecies biofilms must consider the absence of antagonistic relations among the microorganisms. Therefore, under the conditions of the present study, the authors used a co-culture biofilm with proven synergistic behavior among species [37]. Evaluation of a mixed biofilm with antagonist species could generate a false impression of the efficacy of the antimicrobial solutions evaluated. Lastly, analysis of the toxicity of the substance is still necessary for making it feasible to design clinical studies.

Peri-implant biofilm was mimicked by cell adhesion to titanium surfaces, using a previously described methodology [23, 38]. In spite of being an in vitro model, the comparison between mono-species of *C. albicans* and co-culture biofilms of *C. albicans* and *C. glabrata* allowed the authors to evaluate potential of chemical agents involved in the control of peri-implantitis biofilms. Ideally, such agents may have diffusion capacity along the extracellular matrixes and have an antimicrobial effect against a larger number of cells. Therefore, Brazilian red propolis hydroalcoholic demonstrated these properties, confirming the potential use of this substance for the treatment of peri-implantitis.

## Conclusion

Brazilian red propolis demonstrated antifungal activity against *Candida albicans* and *Candida glabrata*, suggesting it to be an alternative for the treatment of peri-implantitis. The results of this study may direct further investigations into the use of this natural substance. Future studies should explore these antimicrobial effect in a complex biofilm and in vivo.

## Data Availability

The datasets used and/or analysed during the current study are available from the corresponding author on reasonable request.

## Abbreviations

ATCC: American Type Culture Collection
CFU/mL: Colony forming unit per milliliter
MTT: Methyl-tretrazolium salt
SDH: Succinate dehydrogenase
Sa: Surface roughness area
MIC: Minimum inhibitory concentration
